# Porcine Dendritic Cells as an In Vitro Model to Assess the Immunological Behaviour of *Streptococcus suis* Subunit Vaccine Formulations and the Polarizing Effect of Adjuvants

**DOI:** 10.3390/pathogens6010013

**Published:** 2017-03-22

**Authors:** Léa Martelet, Sonia Lacouture, Guillaume Goyette-Desjardins, Guy Beauchamp, Charles Surprenant, Marcelo Gottschalk, Mariela Segura

**Affiliations:** 1 Laboratory of Immunology of the Swine and Poultry Infectious Diseases Research Centre (CRIPA), Faculty of Veterinary Medicine, University of Montreal, Saint-Hyacinthe, QC J2S 2M2, Canada; lea.martelet@live.fr (L.M.); guillaume.goyette-desjardins@umontreal.ca (G.G.-D.); 2 Laboratory of *Streptococcus suis*, CRIPA, Faculty of Veterinary Medicine, University of Montreal, Saint-Hyacinthe, QC J2S 2M2, Canada; sonia.lacouture@umontreal.ca (S.L.); marcelo.gottschalk@umontreal.ca (M.G.); 3 Biostatistics Office, Faculty of Veterinary Medicine, University of Montreal, Saint-Hyacinthe, QC J2S 2M2, Canada; guy.beauchamp@umontreal.ca; 4 F. Ménard Inc., 251 Route 235, Ange Gardien, QC J0E 1E0, Canada; csurprenant@fmenard.com

**Keywords:** swine, dendritic cells, adjuvants, vaccines, *Streptococcus suis*, cytokines, Poly I:C, saponins, alum, water-in-oil emulsions

## Abstract

An in vitro porcine bone marrow-derived dendritic cell (DC) culture was developed as a model for evaluating immune polarization induced by adjuvants when administered with immunogens that may become vaccine candidates if appropriately formulated. The swine pathogen *Streptococcus suis* was chosen as a prototype to evaluate proposed *S. suis* vaccine candidates in combination with the adjuvants Poly I:C, Quil A ®, Alhydrogel ®, TiterMax Gold ® and Stimune ®. The toll-like receptor ligand Poly I:C and the saponin Quil A ® polarized swine DC cytokines towards a type 1 phenotype, with preferential production of IL-12 and TNF-α. The water-in-oil adjuvants TiterMax Gold ® and Stimune ® favoured a type 2 profile as suggested by a marked IL-6 release. In contrast, Alhydrogel ® induced a type 1/type 2 mixed cytokine profile. The antigen type differently modified the magnitude of the adjuvant effect, but overall polarization was preserved. This is the first comparative report on swine DC immune activation by different adjuvants. Although further swine immunization studies would be required to better characterize the induced responses, the herein proposed in vitro model is a promising approach that helps assessing behaviour of the vaccine formulation rapidly at the pre-screening stage and will certainly reduce numbers of animals used while advancing vaccinology science.

## 1. Introduction

*Streptococcus suis* is an encapsulated bacterium and an important cause of disease in swine, including meningitis, septicemia with sudden death, endocarditis and arthritis. In addition to the important economic losses to the swine industry, *S. suis* is also an emerging zoonotic pathogen [1,2]. *S. suis* was originally classified into 35 serotypes based on the capsular polysaccharide (CPS) antigenicity, although the current taxonomical situation has been recently revised [3]. Nevertheless, serotype 2 remains the most virulent and frequent capsular type, isolated worldwide from both swine and humans [4]. A high genetic and phenotypic diversity of *S. suis* strains within serotype 2 is reported according to geographical distribution [4]. Pigs are affected generally between 5 and 10 weeks of age, when passive immunity provided by colostrum decreases [5,6]. The pathogenesis of *S. suis* infection is not fully understood. In swine, the main port of entry for *S. suis* is the upper respiratory tract [7]. Subsequently, this pathogen can overthrow the immune system, through an arsenal of virulence factors, including the CPS [8], to cause acute septicemia that may lead to septic shock or different clinical outcomes depending on the colonized organ via mechanisms that are only partially elucidated to date [9].

So far, bacterins (either commercial or, more commonly, autogenous vaccines) have been used in the field to prevent *S. suis* disease with controversial results, and demonstrating only homologous protection [10]. Other strategies, such as live-attenuated or sub-unit vaccines, have been experimentally tested. The use of live avirulent strains gave inconsistent results and may present some safety concerns (zoonosis) [10]. More recently, research has focused on *S. suis* surface proteins as potential sub-unit vaccine candidates. One candidate is enolase, a *S. suis* 52-kDa surface protein that it is expressed by *S. suis* of all serotypes and is involved in adhesion to extracellular matrix components. Furthermore, enolase is a highly conserved protein, and anti-enolase antibodies have been detected in convalescent pig sera [11]. Nevertheless, immunization studies showed that protection conferred by enolase seems to depend on the adjuvant used in the vaccine formulation [12,13,14]. Similarly, it was recently reported that generation of protective antibodies after immunization with *S. suis* CPS as antigen was restricted to CPS conjugation to an immunogenic carrier protein and the use of emulsifying adjuvants [15]. CPS is a promising vaccine candidate because it is the antigen at the base of serotyping and thus can confer universal protection against all strains within the same serotype [10].

The development of the immune response starts with activation of different types of cells involved in the innate immunity including antigen presenting cells (APCs), such as dendritic cells (DCs). DCs are powerful APCs and strongly influence the outcome of the ensuing immunological events. After the capture of antigens, DCs undergo a maturation process and release several cytokines playing a key role in the development of the adaptive immune response. Indeed, mature DCs migrate to adjacent lymphoid organs where they activate T cells. The action of different cytokine pathways drives differentiation of T cells into distinct subtypes, such as Th1 and Th2, which ultimately influence the class of humoral immune responses elicited by B cells. For example, IL-12 and TNF-α are associated with the production of Th1 cells and type 1 IgG subclasses (IgG2a, IgG2b, IgG2c and IgG3, in mice), whereas IL-6 and other Th2 cytokines contribute to type 2 IgG subclass (IgG1) production [16,17,18]. Indeed, IL-6 promotes IL-4-induced Th2 differentiation and inhibits IL-12-induced Th1 differentiation [19]. Different IgG subclasses fulfill distinct biological functions, and thus their relative production is an important consideration when evaluating protection by a vaccine candidate [20,21]. For example, in mice, a dominant type 1 antibody response is associated with opsonophagocytosis and clearance of extracellular encapsulated bacteria, like *S. suis* [10,22,23]. In pigs, the concept of type 1/type 2 IgG subclasses is not completely documented. Crawley et al. [24] determined that porcine Th1 cytokines, IFN-γ and IL-12, induce an IgG2 profile in pigs and that porcine IgG2 activates complement more efficiently than IgG1. In this regard, recent studies suggested that swine IgG2 antibodies are more capable of opsonization than IgG1 antibodies [10,25].

Adjuvants can dramatically influence the vaccine-induced antibody response including bias to type 1 or type 2 responses, which may have a significant effect on the protective efficacy of a vaccine [26,27]. For instance, *S. suis* protein candidates may behave as protective [14,22] or non-protective [12,28] immunogens at least in part depending on the adjuvant. Compared to human medicine, a wider range of adjuvants has been successfully used in commercial vaccines for animals and several new technologies are currently in preclinical development (reviewed in [29,30]). These adjuvants include traditional mineral salt-based adjuvants, oil-in-water/water-in-oil emulsions and saponins. In addition, pathogen-associated molecular patterns, such as Toll like receptor (TLR) ligands, are well-documented immunomodulators that are increasingly recognized as critical components of many modern vaccines. However, the potential of these adjuvants (either traditional or modern ones) to drive the desired type of adaptive immune response when combined to an immunogenic vaccine candidate is generally unknown or poorly characterized in veterinary medicine. Very frequently, the choice of adjuvants is based on theoretical assumptions of their expected type 1 or type 2 polarizing properties, or on their previous use in vaccine formulations in the veterinary or swine field. 

Consequently, in the present study, an in vitro porcine bone marrow-derived dendritic cell (bmDC) culture was used as a model for evaluating immune polarization induced by different adjuvants when administrated with immunogens that have the potential to become vaccine candidates if appropriately formulated. We hypothesize that this in vitro model can reduce the number of animals used in pre-clinical trials by providing fundamental immunological knowledge on selected vaccine formulations (from several possible ones) that would deserve further analysis in animal trials. To develop this in vitro system, different *S. suis* antigens (enolase, CPS and its conjugated form) were used to evaluate whether the in vitro bmDC culture can differentiate the immunogenic potential of the antigen in combination with different polarizing adjuvants.

## 2. Results

### 2.1. Characterization of BmDCs Differentiated by in House-Prepared Porcine GM-CSF

Expression of cell surface markers of swine bmDCs was assessed by FACS after eight days of culture with the in house-produced porcine granulocyte-macrophage colony-stimulating factor (pGM-CSF) supernatant in comparison to commercial swine recombinant (r)GM-CSF (Table 1). Cells were shown to be MHC-I^+^, MHC-II^+^, SWC3^+^, CD1^+^, CD16^+^, CD14^+^, CD11R1^−^ and CD4a^low/−^, as previously described [31,32]. Dose-response studies showed that final 1/50 dilution of pGM-CSF supernatant was optimal for the generation of bmDCs in vitro (data not shown). No significant differences were observed in bmDC phenotype obtained by culturing in the presence of in house-prepared pGM-CSF compared to that obtained when using commercial rGM-CSF (Table 1). These results suggest that the use of a CHO-K1/pGM-CSF stable cell line to generate the growth factor is an efficient and low-cost alternative to use of commercial rGM-CSF, especially when large numbers of bmDCs are required. Indeed, the total yield of differentiated bmDCs per animal was of approximately 1 × 10^8^ bmDCs.

### 2.2. Dose Response and Cytotoxicity of Selected Adjuvants

When using adjuvants to activate immune cells in a closed system, as in the case of a culture well, the main challenge is to find the optimal concentration that will preserve the adjuvant properties but with the least possible toxic effect towards cells. To this aim, bmDCs were activated with different concentrations of the following adjuvants: Poly I:C (a synthetic double stranded RNA that activates multiple elements of the host defense, mainly through recognition by TLR3 [33]); Quil A ® (a saponin belonging to the group of glycosides commonly found in plants that have been tested and commercialized for use in animals [29]); and Alhydrogel ® (an aluminium-based adjuvant, generically referred to as “alum”, already described in 1926 and currently the most widely used in humans and animals [34]). In addition, different added volumes of the emulsifying adjuvants TiterMax Gold ® and Stimune ® were evaluated. TiterMax Gold ® is a water-in-oil adjuvant consisting of squalene as metabolizable oil, sorbitan monooleate 80 as an emulsifier, CRL8300 block copolymer and microparticulate silica as stabilizers. TiterMax Gold ® was developed as a superior alternative to Freund’s adjuvant providing comparable titers with fewer injections and less undesired reactivity in mice [35]. Stimune ® (also known as Specol) is a water-in-oil adjuvant composed of purified and defined mineral oil (Marcol 52) with Span 85 and Tween 85 as emulsifiers [36].

Cell toxicity and cytokine production were assessed in parallel. The concentration of Poly I:C (50 μg/mL) was chosen according to Mussa et al. [37], and induced very low cell toxicity (Figure S1B). In spite of low to moderate toxicity, the highest production of cytokines was obtained with 5 μg/mL of Quil A ®, 50 μg/mL of Alhydrogel ®, and 100 μL/well of TiterMax Gold ® or Stimune ® emulsions (Figure S1A,B). Induction of some level of toxicity is an adjuvant feature also observed in vivo [36,38] that might also contribute to the activation of immune cells at the site of the injection. Regardless of this effect in our in vitro culture system, a clear enhancement and polarization of DC activation is observed when combining the adjuvants with an antigen (see below), suggesting that culture conditions were adequately standardized.

### 2.3. Adjuvants Intensify the BmDC Activation Potential of a Protein Antigen

**Figure 1:**
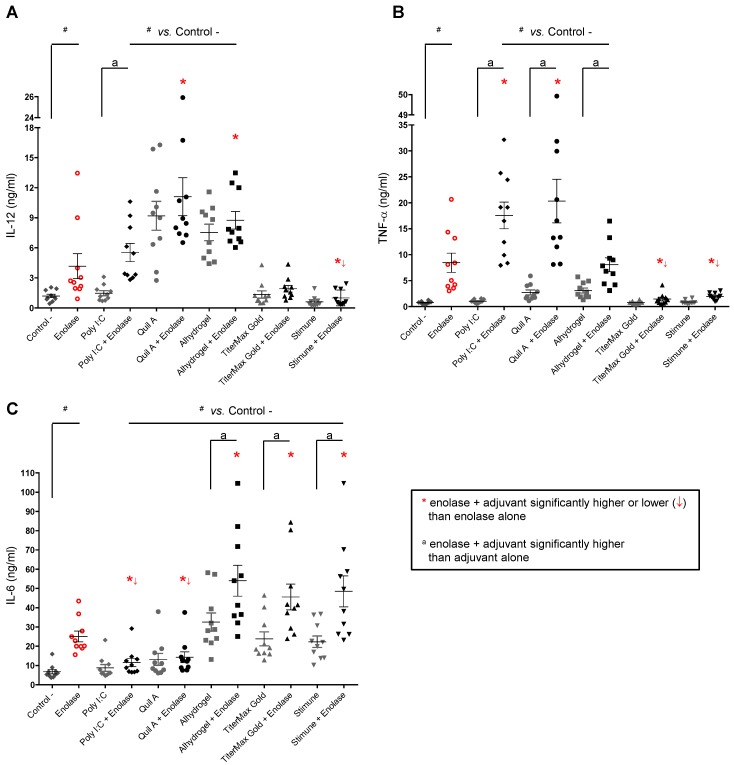
Cytokine production by bmDCs is differentially modified by enolase in combination with different adjuvants. BmDCs derived from 10 different animals were incubated with enolase (50 μg/mL – final concentration for all conditions), alone or in combination with the adjuvants Poly I:C (50 μg/mL), Quil A ® (5 μg/mL), Alhydrogel ® (50 μg/mL), TiterMax Gold ® (100 μL/well of adjuvant–antigen emulsion) or Stimune ® (100 μL/well of adjuvant–antigen emulsion). Adjuvants alone were also evaluated. Cells incubated with medium served as negative controls (-). Cytokine levels (at 24 h of incubation) were evaluated by ELISA. Data of individuals are presented including mean ± SEM in ng/mL (*n* = 10). ^#^ *p* < 0.0001–0.0005, denotes values that are significantly higher than control (-). * *p* < 0.0001, denotes values obtained with enolase in combination with each adjuvant that are significantly higher or lower than enolase alone. ^a^ *p* < 0.0001–0.001 denotes values obtained with enolase in combination with each adjuvant that are significantly higher than the respective adjuvant alone.

To determine the ability of adjuvants to increase the activation of bmDCs induced by the vaccine candidate enolase, each adjuvant alone or in combination with enolase (at the selected doses; Figures S1 and S2) were incubated with bmDCs during 24 h, and then the production of type 1 or type 2 signature cytokines (namely IL-12, TNF-α and IL-6) was measured. As shown in Figure 1, enolase alone induced significant levels of the three evaluated cytokines when compared to non-activated cells (used as negative control). The adjuvant Poly I:C alone failed to induce significant levels of cytokine production by bmDCs. However, when combined with enolase a synergistic effect on TNF-α and, to a lesser extent, on IL-12 production is observed (Figure 1A,B), whereas levels of IL-6 were reduced (Figure 1C). The adjuvant Quil A ® alone induced high levels of IL-12 production by bmDCs, which was not further increased by the presence of enolase. Nevertheless, a significant synergistic effect is observed between enolase and Quil A ® for TNF-α production (Figure 1A,B). Similarly to Poly I:C, enolase-induced IL-6 production is reduced in the presence of Quil A ® (Figure 1C).

The adjuvant Alhydrogel ® alone induced high levels of IL-12 production by bmDCs, which was not further increased by the presence of enolase. On the other hand, this adjuvant induced low levels of TNF-α production and did not modify those induced by enolase (Figure 1A,B). In contrast to Poly I:C and Quil A ® adjuvants, Alhydrogel ® induced high levels of IL-6 production and synergistically enhanced this cytokine release by enolase-activated bmDCs (Figure 1C).

The emulsifying adjuvants TiterMax Gold ® and Stimune ® alone failed to induce IL-12 and TNF-α release by bmDCs. Moreover, they markedly reduced the production of these two cytokines by enolase-activated bmDCs (Figure 1A,B). Nevertheless, and similarly to Alhydrogel ®, emulsifying adjuvants induced high levels of IL-6 production and showed a synergistic effect on this cytokine release by enolase-activated bmDCs (Figure 1C). 

These results suggest that adjuvants, in combination with enolase, intensify bmDC activation, although different cytokine patterns are generated.

### 2.4. BmDCs Can Distinguish Type 1 vs. Type 2 Adjuvants in Combination with Enolase

**Figure 2:**
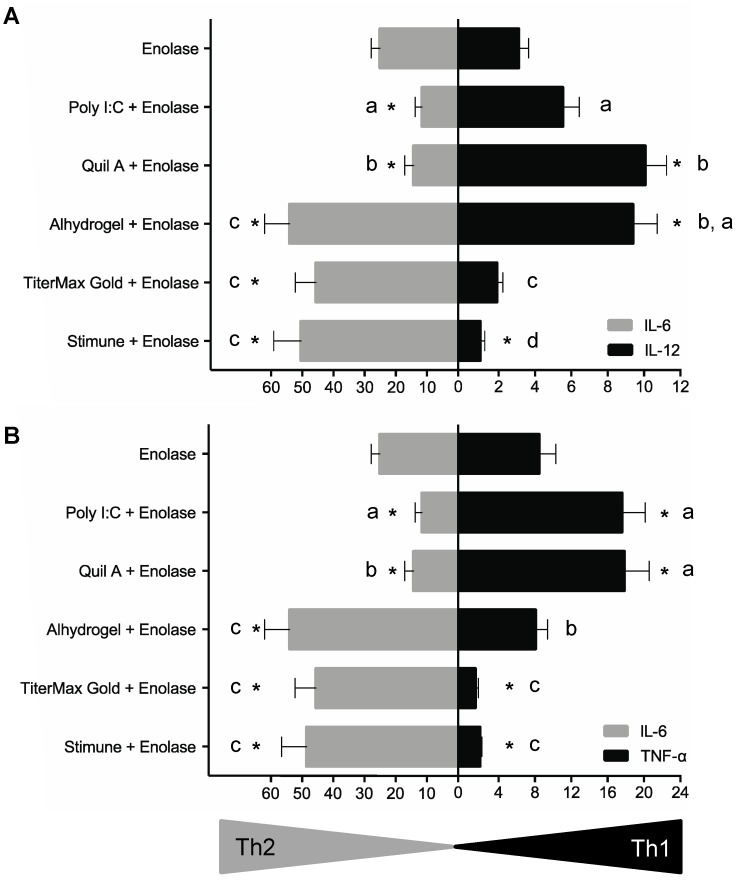
Type 1/type 2 cytokine profiles of bmDCs stimulated with enolase in combination with different adjuvants. Data obtained in Figure 1 using bmDCs derived from 10 different animals and incubated with enolase (50 μg/mL – final concentration for all conditions), alone or in combination with Poly I:C (50 μg/mL), Quil A ® (5 μg/mL), Alhydrogel ® (50 μg/mL), TiterMax Gold ® (100 μL/well of adjuvant–antigen emulsion) or Stimune ® (100 μL/well of adjuvant–antigen emulsion) were analyzed using a linear mixed model to determine the polarizing effect of adjuvants. Data are expressed as mean ± SEM in ng/mL (*n* = 10). * *p* < 0.0001 denotes values obtained with enolase in combination with each adjuvant that are significantly higher or lower than enolase alone. Letters indicate differences between adjuvants in their capacity to induce the different cytokines (*p* < 0.0001–0.0005).

As different patterns of cytokines were observed depending on the adjuvant used in combination with enolase, a type 1 (IL-12 or TNF-α) vs. type 2 (IL-6) profile was tentatively established and statistically analyzed. Results of these analyses are displayed in Figure 2 by either using IL-12/IL-6 or TNF-α/IL-6 comparative expression. Independently of the cytokine combination used for the analyses, enolase alone induced a mixed type1/type 2 cytokine pattern. Poly I:C and Quil A ® biased this response towards a type 1 phenotype by reducing IL-6 production and favoring IL-12 and TNF-α production by bmDCs. Alhydrogel ® amplified enolase-induced IL-6 production, although TNF-α levels were maintained and a strong induction of IL-12 release was also observed, altogether suggesting a mixed type1/type2 profile. In contrast, the emulsifying adjuvants TiterMax Gold ® and Stimune ® significantly diminished enolase-induced production of IL-12 and/or TNF-α and favoured IL-6 production when compared to enolase alone. These latter observations suggest a type 2 bias of bmDC response to enolase with the two emulsifying adjuvants tested.

As shown by the letters in Figure 2, an inter-adjuvant statistical analysis showed that Quil A ® and Alhydrogel ® have the highest capacity to favour IL-12 production by porcine bmDCs. Quil A ® also has the highest capacity to influence TNF-α production, a shared feature with Poly I:C. On the other hand, Alhydrogel ®, TiterMax Gold α and Stimune ® were equally powerful inducers of IL-6.

### 2.5. BmDC Cytokine Response Is Differentially Modulated upon the Chemical Nature of Antigens, Which in Turn Partially Affected the Polarizing Activity of Adjuvants

**Figure 3:**
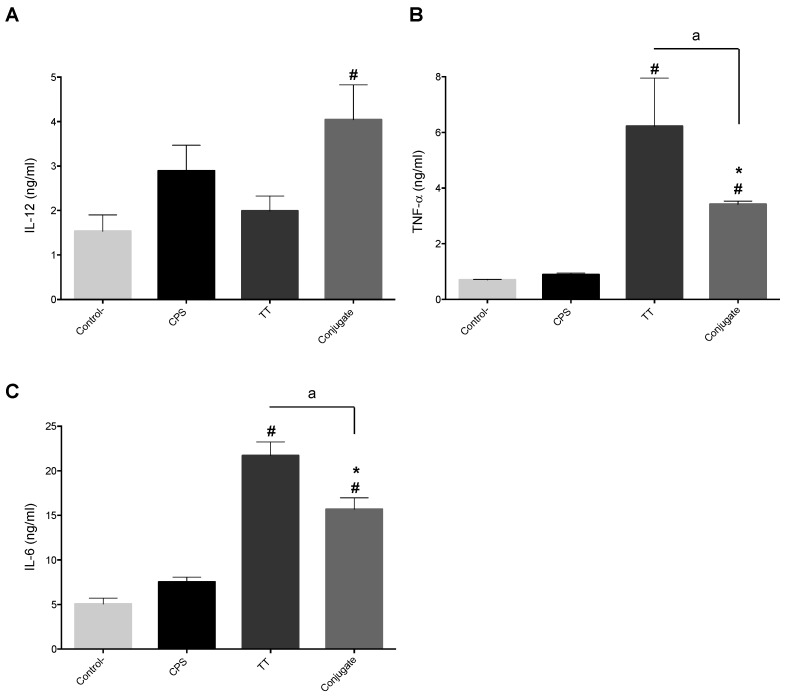
Cytokine production by bmDCs in response to stimulation by capsular polysaccharide (CPS), tetanus toxoid protein (TT) or CPS-TT conjugate. BmDCs (from three different animals) were incubated with CPS, TT, or CPS-TT conjugate at 25 μg/mL. Cells incubated with medium served as negative controls (-). Cytokine levels (at 24 h of incubation) were evaluated by ELISA. Data are expressed as mean ± SEM in ng/mL (*n* = 3). ^#^ *p* < 0.05 denotes values that are significantly higher than control (-).* *p* < 0.01, denotes values obtained with CPS-TT conjugate that are significantly higher than CPS alone. ^a^ *p* < 0.05 denotes values obtained with CPS-TT conjugate that are significantly lower than TT alone.

To determine the ability of bmDCs to discriminate the immunogenic potential of antigens with diverse chemical nature (polysaccharide, protein or polysaccharide-protein), the effects of CPS, tetanus toxoid protein (TT) or a CPS-TT conjugate were evaluated. As expected [39] and due to its polysaccharide nature, CPS alone failed to induce significant levels of cytokine release by porcine bmDCs (Figure 3). The protein TT induced high levels of TNF-α and IL-6, but non-significant levels of IL-12. The CPS-TT conjugate slightly increased IL-12 production when compared to non-stimulated control cells. Compared to CPS alone or control cells, the conjugate also induced higher levels of TNF-α and IL-6. However, levels of these two cytokines were lower than those induced by TT alone, suggesting that the CPS modifies TT activity when the two components are conjugated together. 

**Figure 4:**
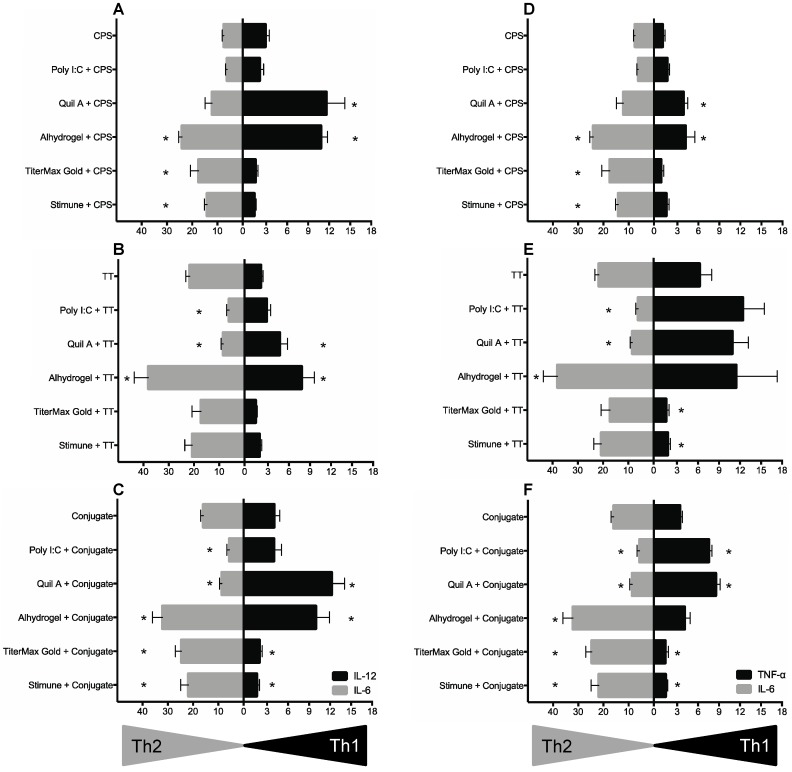
Type 1/type 2 cytokine profiles of bmDCs stimulated with antigens of diverse chemical natures in combination with different adjuvants. BmDCs (from 3 different animals) were incubated with capsular polysaccharide (CPS), tetanus toxoid protein (TT), or CPS-TT conjugate (25 μg/mL – final concentration for all conditions), alone or in combination with the adjuvants Poly I:C (50 μg/mL), Quil A ® (5 μg/mL), Alhydrogel ® (50 μg/mL), TiterMax Gold ® (100 μL/well of adjuvant–antigen emulsion) or Stimune ® (100 μL/well of adjuvant–antigen emulsion). Cytokine levels (at 24 h of incubation) were evaluated by ELISA. Data are expressed as mean ± SEM in ng/mL (*n* = 3). * *p* < 0.05 denotes values obtained with CPS, TT or conjugate in combination with each adjuvant that are significantly higher or lower than the antigen alone.

To determine if the capacity of bmDCs to distinguish the polarizing effect of adjuvants depends on the chemical nature of the antigen or the identity of the antigen, type 1 (IL-12 or TNF-α) vs. type 2 (IL-6) profiles were analyzing using bmDCs activated with CPS, TT, or CPS-TT conjugate in combination with different adjuvants. As shown in Figure 4, in contrast to the results using a protein antigen, Poly I:C failed to overcome CPS unresponsiveness and to polarize the immune response of this polysaccharide antigen. In the case of Quil A ®, no clear polarizing effect on CPS capacity to activate bmDCs was observed, and IL-12 and TNF-α production by bmDCs seemed to be mainly related to the intrinsic capacity of this adjuvant to induce the release of these cytokines (Figure 1 and Figure 4). Similarly, the mixed type1/type2 profile induced by Alhydrogel ® and the type 2 cytokine profile induced by the two emulsifying adjuvants were not modified/amplified in the presence of CPS (Figure 1 and Figure 4). 

On the other hand, a bias towards a type 1 phenotype was observed when either TT or CPS-TT conjugate were combined with Poly I:C or Quil A ®, as previously observed with enolase, with a clear suppression of IL-6 production by bmDCs. Nevertheless, the capacity of these adjuvants to enhance TNF-α and/or IL-12 production by TT-stimulated bmDCs was limited when compared to the effect observed in combination with enolase. Furthermore, Quil A ®-intrinsic capacity to induce production of IL-12 was partially impaired in the presence of TT (*p* < 0.05), but not in the presence of the CPS-TT conjugate (Figure 4). A similar effect on Alhydrogel ®-induced IL-12 production was observed when combined with TT, although a mixed type1/type2 response was still obtained when using Alhydrogel ® + TT or Alhydrogel ® + CPS-TT conjugate formulations (Figure 4). Finally, in spite of a clear suppressive effect on TNF-α production, the emulsifying adjuvants TiterMax Gold ® and Stimune ® failed to provide a type 2 (IL-6) adjuvating effect when formulated with TT, compared to that induced by TT alone. On the other hand, these adjuvants when emulsified with the CPS-TT conjugate promote a bias to a type 2 response, with moderate, but significant, increase in IL-6 production (Figure 4). These results suggest that the amplitude of the adjuvant effect might depend on the chemical nature of the antigen.

## 3. Discussion

The present work developed a model of porcine bmDC in vitro culture for analyzing sub-unit vaccine candidates with previously reported immunogenic potential in combination with various adjuvants for the control of *S. suis* infections. The use of this model provided fundamental knowledge on the polarizing effect of the adjuvant and thus the expected benefits if included in a vaccine formulation. The generated information would potentially contribute to reducing the number of animals used in pre-clinical trials. Furthermore, this study provides for the first time specific information on swine DC immune activation by the adjuvants Quil A ®, Alhydrogel ®, TiterMax Gold ® and Stimune ®. This information is highly significant as species-specific responses might be obtained for a given adjuvant, as described below.

In our porcine bmDC in vitro culture system, Poly I:C and Quil A ® behave as type 1 polarizing adjuvants. Interestingly, Poly I:C lacks intrinsic capacity to induce the release of cytokines (at least those evaluated in this study), but it is able to polarize the cytokine profile induced by the antigen. Similarly, it has been reported that this TLR-ligand possesses weak or no stimulatory ability by itself [37,40], but enhances the release of IL-12 by porcine bmDCs stimulated by the bacterial pathogen *Haemophilus parasuis* [37]. In contrast to swine DCs, human monocyte-derived DCs, purified human CD11c^+^ myeloid DCs, or murine bmDCs produce significant levels of IL-12p40/p70 after Poly I:C stimulation [41,42,43]. In addition, mouse origin DCs also respond with significant levels of TNF-α or IL-6 release after stimulation with this TLR ligand [43,44,45]. In contrast to Poly I:C, Quil A ® alone induces the release of high levels of IL-12 by swine bmDC and, consequently, biases cell activation towards a type 1 profile when combined with an antigen in vitro. Nevertheless, it is unclear whether inter-species differences are also expected in Quil A ® capacity to modulate DC activation in vitro as no studies are available with human or mouse origin DCs.

Several vaccination trials in swine, including one against *S. suis*, reported the use of Quil A ® as adjuvant [22,46,47]. In a field swine immunization study, Quil A ® induced a biased response towards IgG2 directed against *Taenia solium* antigens [46]. Swine immunization with recombinant *S. suis* Sao protein formulated with Quil A ® conferred protection against *S. suis* infection, which was correlated with a predominant IgG2 response [22]. Thus, studies suggest that Quil A ® behaves as a type 1 adjuvant in swine, and our in vitro data supports this concept. Albeit not completely characterized, when used as adjuvant in swine anti-viral vaccines [48,49,50], Poly I:C was suggested to induce a type 1 protective response; results with porcine bmDCs confirm this hypothesis, at least when combined with protein antigens. 

In contrast to swine immunization models, either a type 1 or a mixed type1/type 2 response is generally reported in mouse models when immunizing with Quil A ® or Poly I:C in combination with several antigens [22,51,52,53,54,55], supporting the aforementioned inter-species differences observed in vitro. In this regard, *S. suis* enolase formulated with Quil A ® triggered a balanced type 1/type2 profile in mice [12]. As immunization with enolase + Quil A ® failed to protect mice against a *S. suis* challenge, it would be interesting to evaluate if this formulation has a better protective effect in swine, whereas a more marked bias to a type 1 response would be expected. 

The cellular and molecular pathways involved in ‘alum’ mechanism of action have been extensively studied [34,56]. A significant consideration in the selection of alum as an adjuvant is that alum is universally considered to preferentially support a Th2 immune response, mainly based on mouse models [34,56,57]. Immunization of mice with different *S. suis* immunogenic proteins with alum also suggests a favoured induction of a type 2 antibody response [10]. Fewer studies have analyzed alum-polarizing effects in swine. Although an IgG2 response is also observed in pigs vaccinated with alum-adjuvanted vaccines, a higher IgG1/IgG2 ratio is observed, suggesting a predominant Th2 immune response [58,59]. In vitro, swine DCs responded to alum with a mixed type1/type2 cytokine profile. It has been proposed that alum mainly stimulates a Th2 immune response without affecting the Th1 response [59]. Using different immunization protocols, a study showed that type 1 and type 2 components were present in all protocols, and it was the balance between the opposing cytokines that determined the final outcome of the humoral response in vivo [60]. Thus, the alum polarizing effect remains to be fully characterized in swine models, and, more particularly, for *S. suis* antigens. 

Using the in vitro culture of porcine bmDCs, we showed that the water-in-oil adjuvants TiterMax Gold ® and Stimune ® polarize the immune response towards a type 2 phenotype. Stimune ® has been largely used in veterinary medicine, including swine vaccination trials against *S. suis* [10,15,61,62]. However, the polarizing effect of this adjuvant after swine immunization has been poorly addressed [10]. A recently reported pig vaccination trial with the *S. suis* CPS-TT conjugate prototype adjuvanted in Stimune ® showed a preferential IgG1 isotype switch [15], confirming our in vitro observations with swine DCs. Studies in mice also showed that Stimune ® preferentially polarizes the humoral response towards the IgG1 class when combined with either a peptide antigen [63] or a glycoconjugate vaccine [64]. As indicated above, Stimune ® is a Marcol 52-based emulsion, and, when this mineral oil is used as adjuvant in combination with different *S. suis* immunogenic proteins, it has also been shown to induce a Th2-biased antibody response in mice [10]. In contrast, TiterMax Gold ® is a squalene-based adjuvant which has been reported to induce either mixed Th1/Th2 or Th2-polarized humoral responses in mice [15,65,66]. Squalene based oil-in-water emulsions (MF59 and AS03) licensed for human use also stimulate a mixed Th1/Th2 cell phenotype [26]. Data on the adjuvant effect of squalene-based emulsions in swine (in vitro or in vivo) are scarce; however, a study suggested that an AS03-based vaccine might induce a Th2 response in swine [67], supporting our hypothesis that TiterMax Gold ® polarize swine DC responses towards a type 2 phenotype. Further studies in swine with these types of emulsions will provide more information on their adjuvant effect. 

In a vaccine formulation, not only the adjuvant but also the antigen has the potential to modulate DC functions and the overall adaptive immune response generated by the vaccine. In this study, the polarizing effect of the adjuvants was similar when combined with two different proteins or with a glycoconjugate. However, the magnitude of the response varies, highlighting the need to carefully evaluate the intrinsic properties of the antigen when choosing the adjuvant to be incorporated in the vaccine formulation. Similarly to our in vitro results with DCs, mouse immunization studies suggested that the effects of the adjuvants might be antigen-dependent [56,66]. Finally, we demonstrated in vitro that different adjuvants were unable to overcome the poor immunogenicity of purified *S. suis* serotype 2 CPS, a finding that was confirmed in mouse immunization studies [15].

Our data also showed that the overall DC activation might be the result of a simple additive effect or a synergistic effect between the antigen and the adjuvant, or an effect induced by the adjuvant component alone. In addition, when a bias is imposed by the adjuvant, there may be a suppressive effect over a cytokine with consequent enhancement of another one. Within a vaccine formulation, the combination of all these features will dictate the final capacity of the vaccine formulation to modulate DC functions. Signaling pathways induced by adjuvants and involved in the different responses observed are complex and out of the scope of our work; nevertheless, our data and those reported in the literature highlight the risk in directly transferring the results observed in other systems (either mouse or human) to swine systems. 

The present work suggests that swine bmDCs are able to discriminate the polarizing effect of adjuvants in combination with different antigens, according to the cytokine profile observed. This in vitro model was also able to distinguish (with respect to the profile and/or magnitude of the cytokine response) the intrinsic activating capacity of antigens with diverse chemical natures. Although results still remain to be validated in vivo, using swine immunizations, this model represents a valuable tool to examine the immunogenic potential of vaccine candidates while also screening for adjuvants favouring the desired immune response. In addition to the importance of the basic immunological knowledge generated by this in vitro test, by pre-screening formulations for likelihood of success in vivo, this model has the potential to promote the “Three Rs” (“Replacement, Reduction and Refinements”) guiding principle for more ethical use of animals. As recently stated by Perrie et al. [68], an adjuvant or vaccine formulation that fails to stimulate DC activation in vitro is not likely to be successful in vivo. Dissecting the interaction of antigens and adjuvants in vitro with professional APCs (such as DCs) is a simple but promising approach that helps to assess the behaviour of a vaccine formulation rapidly. Ultimately, animal testing of leading formulation(s) will be essential [68]. 

## 4. Materials and Methods

### 4.1. Purification of *S. suis* Enolase

Cloning and purification of enolase were performed as previously described [11]. Briefly, the gene coding for enolase was amplified by PCR and was cloned into pET-32a vector (Novagen, Madison, WI, USA). The primers used were the forward primer 5′-TATAA GGATCC TTGTCAATTATTACTGATGTTTACGC-3′, introducing a *Bam*HI site (underlined letters), and the reverse primer 5′-TATA AAGCTT TTATTTTTTCAAGTTGTAGAATGAGTTCAAGCC-3′, introducing a *Hind*III site (underlined letters). PCR reactions were carried out with Phusion ® high fidelity *taq* polymerase (New England Biolabs, Ipswich, MA, USA). PCR amplification conditions consisted of 2 min at 98 °C and 35 cycles of 10 s at 98 °C, 10 s at 56 °C, and 60 s at 72 °C using a Biometra TGradient thermocycler (Göttingen, Germany) and a final elongation step of 5 min. Plasmid was purified by mini-prep (Qiagen, Toronto, ON, Canada). Purified plasmid was digested with *Bam*HI and *Hind*III in order to confirm presence of insert. Automated sequencing was used to check the amplified enolase gene. The verified complete gene was cloned into pET-32a vector using the *Bam*HI and *Hind*III sites. This plasmid contains a His-tag-encoding sequence of about 25 kDa. The plasmid pET-32a-Enolase was introduced into *E. coli* Bl21DE3 for IPTG-inducible expression of recombinant enolase. The protein was purified by His-Bind ® Resin chromatography (EMD Millipore Corp, Billerica, MA, USA) through the His-tagged fusion according to the manufacturer’s protocol. Protein-containing fractions were determined by NanoDrop ® ND-1000 (Thermo-Fisher Scientific, Waltham, MA, USA). The purity of enolase fractions was verified by Western blotting using optimally diluted monospecific rabbit anti-enolase IgG and peroxidase-conjugated goat anti-rabbit (Jackson ImmunoResearch, Baltimore, PA, USA), as previously described [11]. Final protein concentration was measured by Pierce^TM^ BCA Protein Assay kit (Thermo-Fisher Scientific).

### 4.2. Production and Purification of *S. suis* CPS and Its Conjugate

*S. suis* serotype 2 CPS was produced and purified as previously described [39,69]. Preparation of CPS and TT, covalent coupling of CPS to TT and characterization of the obtained glycoconjugate was performed as previously described [15]. Briefly, purified CPS was depolymerized by ultrasonic irradiation to a *M_W_* of 115 kDa, and then 10% of the capsular sialic acids (Neu5Ac) were mildly oxidized by treating with sodium periodate in order to introduce functional aldehydes for subsequent conjugation to TT, used as the carrier protein. The TT monomer was purified by gel filtration chromatography before conjugation. The glycoconjugate was produced using a molar mixture of 2 oxidized CPS chains:1 TT, which was conjugated by reductive amination for 48 h. The conjugation was stopped by the addition of sodium borohydride to reduce the remaining free aldehydes for 1 h. The resulting conjugate was desalted by extensive dialysis against water and lyophilized. To obtain a representative control of unconjugated CPS for this study, a sample of the same oxidized CPS used for conjugation was reduced, desalted and lyophilized in the same manner as the conjugate. Purified TT was also included as control. 

### 4.3. Production of in House pGM-CSF

CHO-K1/pGM-CSF stable cell line was derived from the CHO-K1 cell line (ATCC ^®^ CCL-61^TM^, Manassas, VA, USA). A 459 bp DNA fragment corresponding to the coding region of *Sus scrofa* colony stimulating factor 2 (CSF2/GM-CSF) mRNA (accession number NM_214118) was chemically synthesized (Genescript, Piscataway, NJ, USA). The synthetized DNA fragment was then cloned into the mammalian expression vector pcDNA3.1(+) using *Eco*RI (upstream) and *Xho*I (downstream) as enzymes to make the expression construct. The final expression construct was sequenced (sequencing primer TGGGAGGTCTATATAAGCAGAG) and was 100% accurate with CSF2/GM-CSF template sequence. CHO-K1 cells were then transfected with this final construct encoding pGM-CSF and stable clones were obtained by selection using geneticin at 600 μg/mL (G418, Gibco, Invitrogen, Thermo-Fisher Scientific). A number of positive clones were selected for further evaluation after culturing for 10 passages. Based on Western blot analysis with monoclonal anti-porcine GM-CSF antibody (MAB711; R&D systems, Mineapolis, MN, USA), clones 10, 25 and 30 were selected for final use. For pGM-CSF production, CHO-K1/pGM-CSF cells were suspended at 5 × 10^5^ cellules/mL in DMEM F12 supplemented with 10% heat-inactivated fetal bovine serum (FBS), 2 mM L-Glutamine and 600 μg/mL of geneticin (600 μg/mL). All reagents were from Gibco. Cells were initially cultured in T25 flasks and then sub-cultured in T75 flasks (Sarstedt, Nümbrecht, Germany) at 37 °C in a 5% CO_2_ incubator for approximately two days until confluence. For sub-culturing, cells were washed with a 2% of EDTA-PBS solution and detached with 0.25% of trypsin for 1 min. For final pGM-CSF production, cells were cultured in T75 flasks in complete medium without geneticin for five days. Finally, supernatants were recovered and centrifuged twice at 830 *g* for 10 min at room temperature. The supernatant was recovered, aliquoted and stored at −80 °C.

### 4.4. Animals and Isolation of Porcine Bone Marrow Cells

Bone marrow cells were obtained from 6–8 week-old piglets (*n* = 10). The animals originated from a herd free of major important diseases such as porcine reproductive and respiratory syndrome, enzootic pneumonia due to *Mycoplasma hyopneumoniae* and clinical disease related to porcine circovirus. The herd did not have any episode of acute disease related to *S. suis* when the samples were taken. All experiments involving animals were conducted in accordance with the guidelines and policies of the Canadian Council on Animal Care and the principles set forth in the Guide for the Care and Use of Laboratory Animals and approved by the Animal Welfare Committee of the University of Montreal (protocol # 2016-Rech-1570). Bone marrow was aseptically removed from the femurs of ten different animals and separately processed using endotoxin-free solutions and materials. Briefly, after removal of muscle tissue, femurs were sliced and stirred in 1 L of PBS for 2 h at room temperature. The PBS suspension containing released bone marrow cells was recovered, filtered through gauzes and centrifuged at 250 *g* for 10 min at 4 °C. After red blood cell lysis (eBioScience, San Diego, CA, USA), cells were washed, filtered through a 40 μm-cell strainer (BD Falcon^TM^, Bedford, MA, USA), and resuspended at approximately 1–3 × 10^7^ cells/mL in a cryopreservation solution containing 95% of FBS and 5% of dimethylsulfoxyde (DMSO) (Sigma-Aldrich, St Louis, MO, USA) and stored in liquid nitrogen until use.

### 4.5. Generation of Porcine BmDCs

Bone marrow cells were thawed and resuspended in RPMI 1640 medium supplemented with 10% heat-inactivated FBS, 2 mM L-glutamine, 10 mM HEPES, 100 U/mL penicillin-streptomycin, and 1 μg/mL gentamycin. All reagents were from Gibco. Complete medium was complemented with either 1/50 dilution of pGM-CSF supernatant or with 100 ng/mL commercial porcine rGM-CSF (R&D system), as previously described [32]. Then, 2.5 × 10^6^ cells/well were cultured in 6 well-tissue culture plates (Falcon ®, Corning, Tewksbury, MA, USA) for 8 days at 37 °C in a 5% CO_2_ incubator and were fed on days 3 and 6 with fresh complete medium. On day 8, cells were harvested, washed, and used as immature DCs for the studies. DC phenotype and purity was confirmed by FACS as described below.

### 4.6. DC Phenotype Analysis by FACS 

Obtained porcine bmDCs were phenotypically characterized by the following markers: MHC-I, MHC-II, CD4a, CD16, CD14, CD11R1, SWC3 and CD1, as previously described [32,70]. Commercially available monoclonal antibodies from AbD Serotec (Kidlington, Oxford, United Kingdom) were used to detect swine MHC-I (clone JM1E3), MHC-II (clone 2E9/13), CD16 (clone G7), and CD4a (MIL17). Monoclonal antibody against swine CD11R1 (clone MIL4) and CD14 (clone MIL2) were from Bio-Rad (Kidlington, Oxford, United Kingdom). Monoclonal antibody against swine CD1 (clone 76-7-4) was from Abcam Inc (Cambridge, MA, USA). A hybridoma specific for SCW3 was used (clone 74.22-15A; ATCC HB-142.1). Antibodies against MHC-I, MHC-II, CD16, CD1 and CD4a were conjugated to FITC. A PE-conjugated goat anti-mouse IgG (Leinco Technologies Inc, St Louis, MO, USA) was used for labelling CD11R1, CD14 and SCW3. The staining and FACS analysis were performed as previously described for swine bmDCs [32] using a FACS BD Accuri C6 Cytometer (BD Biosciences, San Jose, CA, USA).

### 4.7. In Vitro BmDC Stimulation Assay

Bone marrow DCs were resuspended at 10^6^ cells/ml in complete medium and plated into 24 well-culture plate (Falcon ®). Then, different activators were added. Four different antigens were used: enolase at 50 μg/mL (the concentration was chosen based on a preliminary dose response study, see Figure S2); CPS at 25 μg/mL; TT at 25 μg/mL; CPS-TT conjugate at 25 μg/mL (these concentrations were chosen based on [15]). Several categories of adjuvants were evaluated: the TLR-ligand Poly I:C (Novusbio, Oakville, ON, Canada), was added at 50 μg/mL; the saponin Quil A ® (Brenntag Biosector, Frederikssund, Denmark), was added at 5 μg/mL; Alhydrogel 2% ® (Brenntag Biosector), composed of aluminum hydroxide, was added at 50 μg/mL; the water-in-oil emulsion TiterMax Gold ® (Cytrx Corporation, Norcross, GA, USA) emulsified 1:1 (*v/v*) with antigens and the water-in-oil emulsion Stimune ® (Prionics, Lelystad, The Netherlands) emulsified 5:4 (*v/v*) with antigens were added at 100 μL/well to culture plates. Concentrations of different adjuvants were chosen upon a preliminary selection based on the literature [12,15,22,37,63,71,72,73,74] and finally established by a dose response study of toxicity levels and cytokine production (Figure S1). The preparation of adjuvant–antigen mixtures followed the manufacturer’s recommendations. To avoid any influence of possible endotoxin contamination during cell stimulation assays, polymyxin B sulfate (Sigma) at 20 μg/mL was added to the cultures. At 24 h of incubation, supernatants were collected for cytokine quantification by ELISA, as described below. 

### 4.8. Cytokine Quantification by ELISA and Cell Toxicity Test 

Levels of IL-12p40, TNF-α and IL-6 in cell culture supernatants were measured by sandwich ELISA using pair-matched antibodies from R&D Systems or Invitrogen according to the manufacturer’s recommendations. Twofold dilutions of recombinant porcine cytokines were used to generate the standard curves. Sample dilutions giving optical density readings in the linear portion of the appropriate standard curve were used to quantify the levels of each cytokine. Cell toxicity induced by different adjuvants was evaluated by measuring release of lactate dehydrogenase enzyme (LDH) with the CytoTox96 Non-Radioactive Cytotoxicity Assay (Promega, Madison, WI, USA) as previously described [75].

### 4.9. Statistical Analysis

Data were transformed using the logarithm base 10 to normalize distributions. A linear mixed model was used to compare the various treatments. The random factor was the animal and the fixed factor was treatment. The model also included uneven variances for the various treatments. A priori contrasts were performed to compare pairs of means adjusting the alpha level for each comparison using the Benjamini–Hochberg sequential adjustment procedure. The nominal alpha level was set at 0.05 throughout. Statistical analyses were carried out with SAS v.9.4 (Cary, NC, USA). This method was applied for analysis of data displayed in Figure 1 and Figure 2 (*n* = 10). All other data were analyzed for significance using a Student’s unpaired *t*-test, SigmaPlot (version 11.0, Systat Software, San Jose, CA, USA). A *p* value < 0.05 was used as a threshold for significance.

## 5. Conclusions

In conclusion, use of in vitro methods as a replacement for animal models at the pre-screening stages will certainly reduce numbers of animals used while advancing the vaccinology science. This approach can be used to test other *S. suis* vaccine candidates and accelerate the design of promising sub-unit vaccines against this pathogen. 

## Figures and Tables

**Table 1:** Surface phenotype of bmDCs after eight days of differentiation using either in house-produced porcine (p)GM-CSF supernatant or commercial recombinant (r)GM-CSF.

Marker	pGM-CSF Supernatant (% Positive Cells) ^1^	rGM-CSF Commercial (% Positive Cells) ^1^
MHC-I	89 ± 6	90 ± 7
MHC-II	85 ± 6	86 ± 9
SCW3	88 ± 4	86 ± 5
CD1	69 ± 3	67 ± 5
CD16	89 ± 2	93 ± 2
CD14	79 ± 2	84 ± 3
CD4a	14± 2	9 ± 5
CD11R1	4 ± 1	2 ± 1

^1^ Mean ± SEM of four independent experiments using the supernatants of three CHO-K1/pGM-CSF stable cell line clones or from three independent experiments using commercial swine rGM-CSF.

## Supplementary Materials

The following are available online at www.mdpi.com/2076-0817/6/1/13/s1; Figure S1: Dose-response and toxicity trials to select optimal adjuvant dose for in vitro bmDC studies, Figure S2: Dose-response to select optimal enolase concentration for in vitro bmDC studies.
