# Supplementary material for: Porcine Dendritic Cells as an In Vitro Model to Assess the Immunological Behaviour of Streptococcus suis Subunit Vaccine Formulations and the Polarizing Effect of Adjuvants
Source: Pathogens. 2017 Mar 22;6(1):13. doi: 10.3390/pathogens6010013 (PMC5371901; doi:10.3390/pathogens6010013)
Supplement: Supplementary file 1 [file pathogens-06-00013-s001.pdf]

pathogens

MDPI

Supplementary Material: Porcine Dendritic Cells as an In Vitro Model to Assess the Immunological Behaviour of Streptococcus suis Subunit Vaccine Formulations and the Polarizing Effect of Adjuvants

Léa Martelet, Sonia Lacouture, Guillaume Goyette-Desjardins, Guy Beauchamp, Charles Surprenant, Marcelo Gottschalk and Mariela Segura

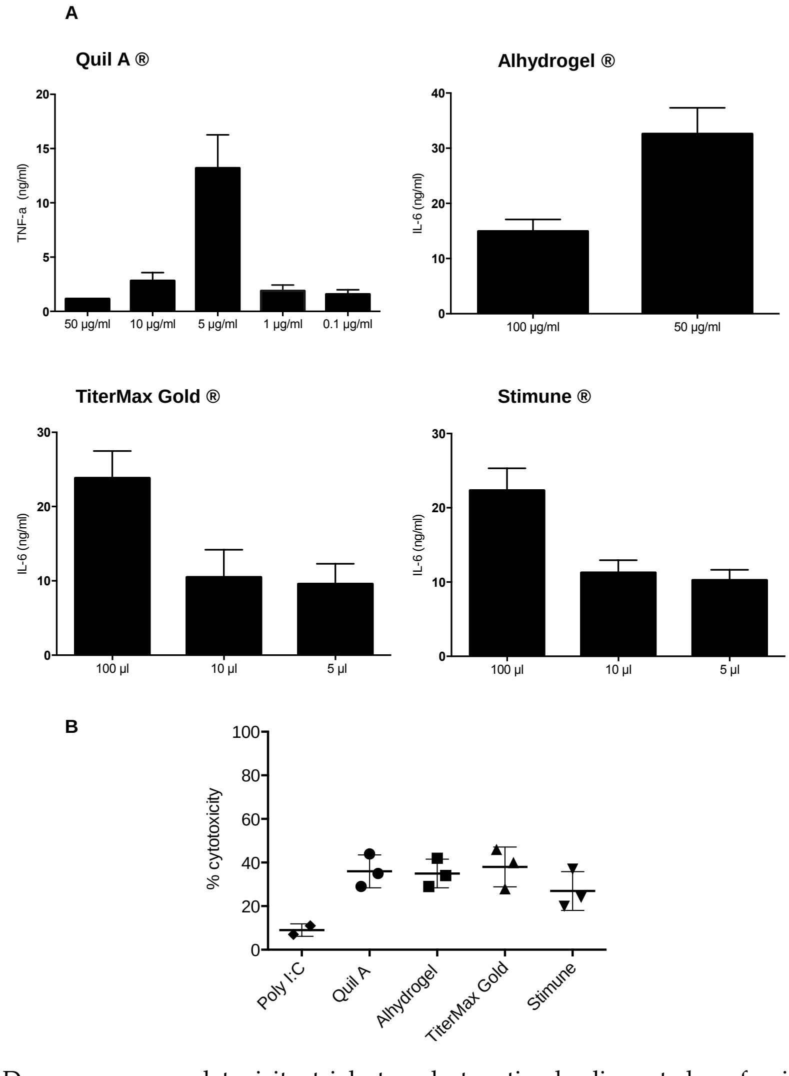

Figure S1. Dose-response and toxicity trials to select optimal adjuvant dose for in *vitro* bmDC studies. (**A**) BmDCs derived from 3 different animals were incubated with different concentrations of adjuvants and cytokine levels in cell culture supernatants were measured by ELISA (data expressed as ng/ml). The adjuvant Poly I:C alone failed to induce significant levels of cytokine production by bmDCs and it was thus not included in Panel A. Representative cytokines were chosen (upon the type 1/type 2 profiles) for other adjuvants. (**B**) Cytotoxicity was evaluated by the lactate dehydrogenase (LDH) enzyme test after bmDC activation with adjuvant concentrations which gave the highest production of cytokine in (**A**), that is: Quil A®: 5 µg/mL; Alhydrogel®: 50 µg/ mL; TiterMax Gold®: 100 µL/well of emulsion; and Stimune®: 100 µL/well of emulsion. Poly I:C was used at 50 µg/ mL. Data of individuals are presented including mean ± SEM (n = 3).

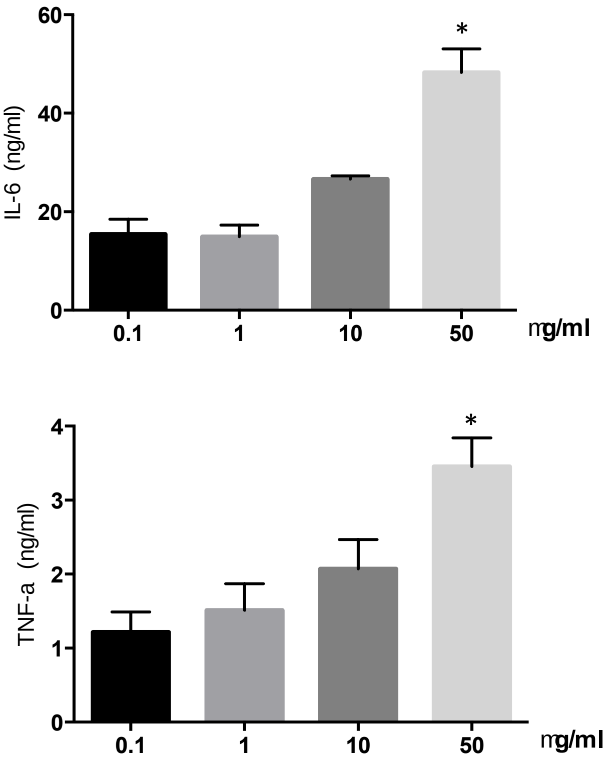

Figure S2. Dose-response to select optimal enolase concentration for *in vitro* bmDC studies. Swine bmDCs derived from 2 different animals were incubated with different concentrations of purified enolase. Cytokine levels in cell culture supernatants were measured by ELISA and data expressed as ng/mL.
